# Single-stranded DNA library preparation uncovers the origin and diversity of ultrashort cell-free DNA in plasma

**DOI:** 10.1038/srep27859

**Published:** 2016-06-14

**Authors:** Philip Burnham, Min Seong Kim, Sean Agbor-Enoh, Helen Luikart, Hannah A. Valantine, Kiran K. Khush, Iwijn De Vlaminck

**Affiliations:** 1 Meinig School of Biomedical Engineering, Cornell University, Ithaca, NY 14853, USA; 2 National Institutes of Health, Bethesda, MD 20892, USA; 3 Division of Cardiovascular Medicine, Stanford University School of Medicine, Stanford CA 94305, USA.

## Abstract

Circulating cell-free DNA (cfDNA) is emerging as a powerful monitoring tool in cancer, pregnancy and organ transplantation. Nucleosomal DNA, the predominant form of plasma cfDNA, can be adapted for sequencing via ligation of double-stranded DNA (dsDNA) adapters. dsDNA library preparations, however, are insensitive to ultrashort, degraded cfDNA. Drawing inspiration from advances in paleogenomics, we have applied a single-stranded DNA (ssDNA) library preparation method to sequencing of cfDNA in the plasma of lung transplant recipients (40 samples, six patients). We found that ssDNA library preparation yields a greater portion of sub-100 bp nuclear genomic cfDNA (*p* 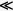 10^−5^, Mann-Whitney U Test), and an increased relative abundance of mitochondrial (10.7x, *p* 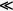 10^−5^) and microbial cfDNA (71.3x, *p* 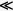
10^−5^). The higher yield of microbial sequences from this method increases the sensitivity of cfDNA-based monitoring for infections following transplantation. We detail the fragmentation pattern of mitochondrial, nuclear genomic and microbial cfDNA over a broad fragment length range. We report the observation of donor-specific mitochondrial cfDNA in the circulation of lung transplant recipients. A ssDNA library preparation method provides a more informative window into understudied forms of cfDNA, including mitochondrial and microbial derived cfDNA and short nuclear genomic cfDNA, while retaining information provided by standard dsDNA library preparation methods.

Cell-free DNA (cfDNA) is quickly finding application as a monitoring tool in pregnancy, cancer and organ transplantation^1^,^2^,^3^,^4^,^5^. cfDNA exists in circulation in many shapes and forms, including fragments of the nuclear genome, the mitochondrial genome and microbial genomes^6^. The predominant type of cfDNA is derived from the nuclear genome and has a fragment size centered around 166 bp, approximately the length of a segment of DNA wound around a histone octamer^7^,^8^. These nucleosomal fragments of cfDNA are readily accessible for sequencing using standard library preparation methods that are based on ligation of dsDNA sequencing adapters. The most commonly used implementations of this method rely on multiple bead-based size-selective steps that eliminate unwanted adapter-dimer products. These methods, although relevant to a wide range of applications, are not sensitive to the full diversity of circulating DNA^9^; in particular shorter fragments, highly degraded fragments and (partially) single-stranded fragments of DNA in circulation remain undetected (Fig. 1).

An interesting parallel exists with genomic analyses of ancient DNA samples, where the target DNA is usually highly fragmented and present in low amounts^10^. Recently, Meyer *et al*. introduced a sequencing library preparation method that is based on single-stranded ligation and demonstrated the method by sequencing of the genome of an extinct archaic human^11^,^12^. Here, we have applied this protocol to sequencing of cfDNA in plasma, motivated by the hypothesis that a library preparation based on single-stranded ligation, is in principle, sensitive to the full diversity of cfDNA in the circulation, including ultrashort (<100 bp) dsDNA, ssDNA and dsDNA with nicks in both strands.

We applied the approach to the analysis of clinical plasma samples collected from lung transplant recipients, prompted by recent studies that have indicated the potential of cfDNA in the monitoring of infection and rejection in solid-organ transplantation^4^,^13^,^14^. To evaluate the performance of the ssDNA library preparation method, we directly compared data of fragment types, lengths and abundance to results from conventional library preparations performed on the same plasma DNA extracts^13^,^15^. Transplant recipients are subject to immunosuppressive therapies that reduce the risk of rejection, but increase their susceptibility to opportunistic infections. Analyses of microbial cfDNA in plasma are therefore particularly relevant in the context of transplantation. Here, we examined the yield of microbial cfDNA that results from the ssDNA and conventional library preparations. Donor specific nuclear genomic cfDNA is present in the circulation of solid-organ transplant patients and is a marker of transplant rejection^2^,^4^,^16^. In this study, we used a single-stranded library preparation to study the properties of donor and recipient specific DNA across a wide length range. To test whether donor specific mitochondrial cfDNA can be found in the circulation of transplant recipients, we directly compared data of cfDNA to reference sequences of amplified mitochondrial genomes obtained from pre-transplant samples. We note that Karlsson *et al*. recently applied a ssDNA ligation protocol to the amplification-free sequencing of cfDNA^17^. These authors, however, did not perform an analysis of fragment lengths and did not examine the presence of mitochondrial and microbial DNA in plasma.

## Results

**Figure 1:**
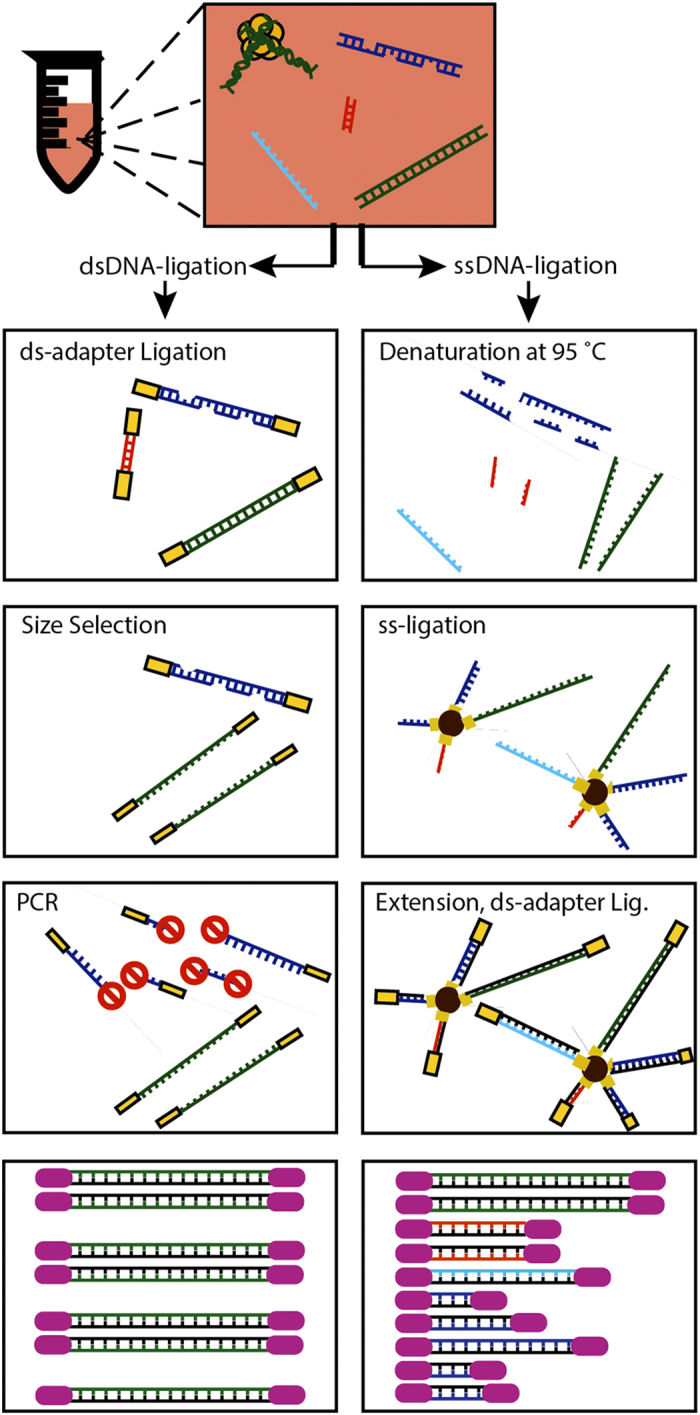
Schematic of sequencing library preparation methods. Schematic illustration of key steps in the dsDNA and ssDNA library preparation protocols used in this work and their sensitivity to different types and forms of circulating cfDNA in plasma. cfDNA in plasma may be single-stranded (light blue), partially single-stranded or nicked (dark blue), short (sub-100 bp, red), or long (super-100 bp, green).

Forty samples of cfDNA extracted from plasma of six double-lung transplant recipients^13^ were analyzed in this study. Libraries were prepared for sequencing using a ssDNA library preparation protocol and sequenced (5.7 ± 1.4 million paired-end reads per sample). Results were compared against sequence data obtained for the same samples following conventional dsDNA library preparation where available (36 matched samples, 18.8 ± 9.1 million paired-end reads per sample). The analysis of matched samples enabled us to assess the effect of different library preparations on measurements of cfDNA. The key distinguishing features of the library preparation protocols and their sensitivity to different forms of cfDNA are schematically represented in Fig. 1.

### Size distribution and abundance of mitochondrial DNA in plasma measured by digital PCR

**Figure 2:**
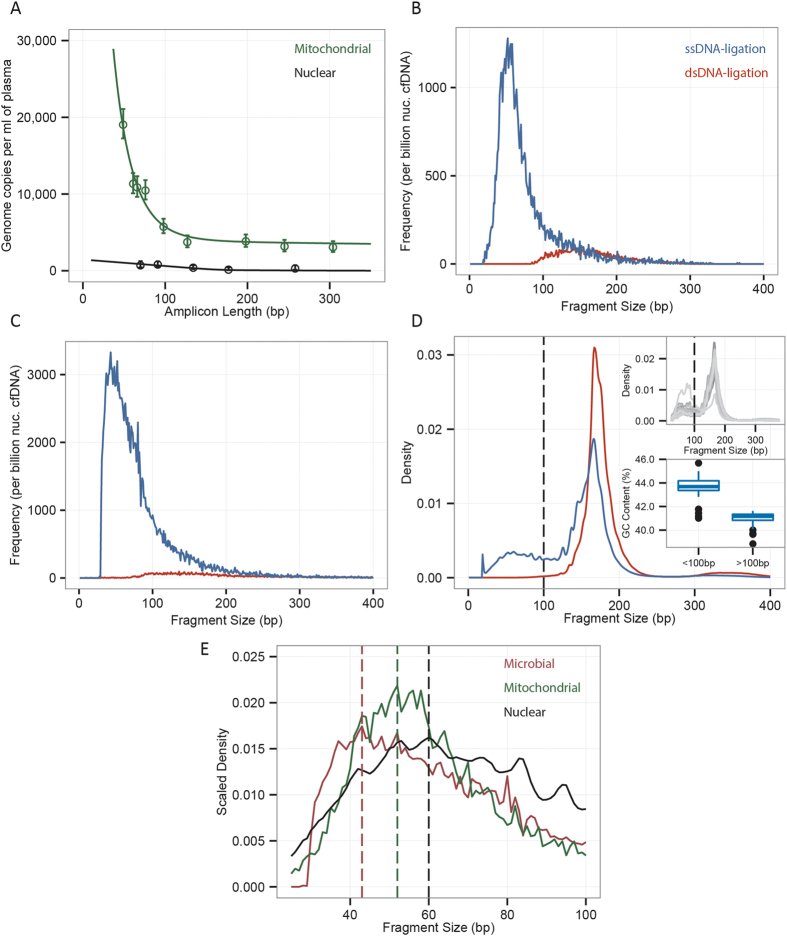
cfDNA fragment length distributions. (**A**) Abundance of mitochondrial (green) and nuclear genomic cfDNA (black) measured by digital PCR assays with different amplicon lengths. Solid lines are model fits (see SI). (**B,C**) Fragment length histograms (frequency relative to total nuclear genomic) measured via sequencing for mitochondrial (**B**) and microbial (**C**) cfDNA following ssDNA (blue) and dsDNA (red) library preparation. (**D**) Density plot of the fragment sizes of nuclear genomic cfDNA measured after ssDNA (blue) and dsDNA (red) library preparation. The inset shows the sample-to-sample variability, as well as the difference in GC content for short (<100 bp) and long (>100 bp) fragments. (**E**) Density (scaled for clarity) of short length (segment lengths <100 bp) mitochondrial, microbial and nuclear genomic cfDNA measured by ssDNA library preparation. Vertical lines highlight most prevalent fragment lengths.

This study was first prompted by a retrospective analysis of sequencing data of cfDNA in plasma of transplant recipients available from a previous study^13^, which revealed a fractional abundance of mitochondrial cfDNA of 2 × 10^−3^%, which is in line with a recent observation^6^, but is low considering that there are 50–4,000 mitochondrial genomes per cell^18^. We used digital PCR (dPCR) assays with varying amplicon length (49–304 bp) to assess the abundance of mitochondrial cfDNA prior to library preparation and compared this to the abundance of nuclear genomic cfDNA (Fig. 2A). The experimental design with variable amplicon lengths provided information about the underlying fragment length distribution^7^: cfDNA is randomly fragmented in plasma, the genomic abundance, as measured by PCR, is therefore expected to decrease monotonically with amplicon length, with a gradient that is a function of the underlying fragment length distribution. These experiments revealed that mitochondrial DNA is more fragmented than nuclear genomic DNA, but present in much greater abundance in plasma (56-fold greater representation, genome equivalents). The consequence of the short fragment size of mtDNA is that conventional dsDNA library preparation protocols, which require multiple bead-based size-selective steps that eliminate unwanted adapter-dimer products, are relatively insensitive to mitochondrial sequences.

### ssDNA library preparation and fragmentation profiles

We next implemented a ssDNA library preparation protocol first described by Meyer *et al*. that does not require size-selective steps that eliminate shorter fragments^11^ (Fig. 1 and Methods). We used paired-end sequencing to determine the fragment lengths of nuclear, mitochondrial and microbial cfDNA (see Methods). Figure 2B–D shows a direct comparison of the fragment length profiles measured after ssDNA and conventional library preparation. We found that cfDNA shorter than 100 bp becomes more accessible for sequencing following ssDNA library preparation. While conventional library preparation resulted in detection of only a few molecules of mitochondrial and microbial cfDNA with length shorter than 100bp, the use of a ssDNA library preparation revealed an abundance of such molecules with lengths between 40 and 100 bp. The lower limit of efficient capture, as shown by the local maxima of the short fragment cfDNA, for the ssDNA library preparation, was 40–60 bp, for all subclasses (mitochondrial, microbial, nuclear genomic cfDNA), pointing to a limit set by the DNA isolation method (Fig. 2E), rather than the library preparation^12^.

The peak in the length profile at 160–167 bp for cfDNA fragments assigned to the nuclear genome (Fig. 2D) is a consequence of the protection of these molecules from degradation by nucleases in the blood through tight association with histones. This property has been reported in previous studies and is observed for both the ssDNA and dsDNA library preparation protocols^19^. A second peak at shorter lengths (<100 bp) is unique to the libraries prepared by single-stranded ligation^15^. The relative proportion of nuclear genomic DNA shorter than 100 bp made up a substantial proportion of nuclear cfDNA (20.54% ± 11.51%). We partitioned non-nucleosomal DNA into two groups, those with length under and over 100 bp, to examine distinguishing features between the two groups. The GC content between the groups differed significantly (Fig. 2D, inset; *p* 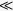 10^−5^, Mann-Whitney U Test); the GC content of the super-100 bp group was 40.9%, while that of the sub-100 bp group was 43.5%. These observations indicate that a considerable amount of nuclear genomic cfDNA is not nucleosome protected and, thus, subject to degradation by nucleases in the blood.

Previous reports suggest that fetal and tumor derived cfDNA are shorter than cfDNA derived from maternal^20^ and normal^6^ tissue, respectively-the sensitivity of the ssDNA library preparation protocol to molecules over a wider length range is therefore a feature that will be useful for applications in prenatal testing and tumor monitoring.

### Improved recovery of mitochondrial and microbial cfDNA

We next examined the coverage of mitochondrial and microbial genomes relative to the nuclear genome for the dsDNA and ssDNA library preparations. We found that the ssDNA library preparation gives rise to an increase in the relative number of mitochondrial sequences in the datasets (10.7 fold mean increase, p 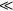 10^−5^, Mann-Whitney U test) and an increase in the relative coverage of the mitochondrial genome (7.22 fold mean increase, p 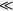 10^−5^, Mann-Whitney U test). This observation is consistent with the greater sensitivity of the ssDNA library preparation to short fragment DNA described above.

**Figure 3:**
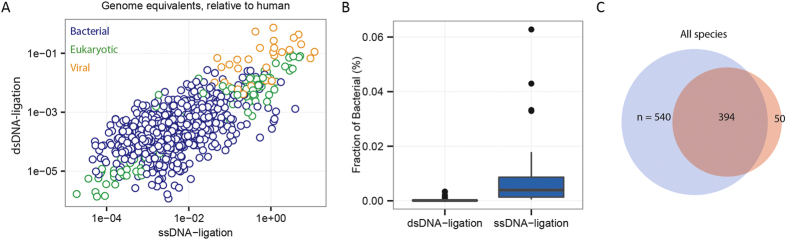
ssDNA library preparation yields greater fraction of non-human cfDNA. (**A**) Comparison of the coverage of microbial genomes relative to the human genome for ssDNA and dsDNA library preparation. Data points are colored by domain of life. (**B**) Yield of bacterial sequences for ssDNA library preparation relative to dsDNA library preparation (74-fold mean increase). (**C**) Venn diagram representation of the number of species uniquely detected following ssDNA library preparation in blue (540/984, 54.9%), species uniquely detected following dsDNA library preparation in red (50/984, 5.1%), and species detected following both protocols (394/984, 40.0%).

To study the efficiency of recovery of microbial derived cfDNA, we estimated the genome coverage of microbes detected across all samples relative to the coverage of the human genome (see Methods). We compared the relative genomic coverage of strains or subspecies detected by both methods in matched samples (n = 36) (Fig. 3A); for example E. coli in lung transplant patient L77 on day 3 was treated as a separate event from E. coli in the same patient on day 2. We examined over 1,100 direct comparisons and found a significant correlation (corr. = 0.6373, Spearman, *p* 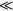 10^−5^) in the relative genomic abundance as measured following ssDNA and dsDNA library preparation (Fig. 3A); the range in the ratio was 0.277x–3950x, indicative that for most species the ssDNA method led to more efficient detection (*p* 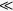 10^−5^; Mann-Whitney U Test). Importantly, library preparation by ssDNA ligation gave rise to a mean 71-fold increase in the relative genomic coverage of microbial species (74-fold for bacteria, which made up 89% of the sampled species comparisons; see SI, Table 1, Fig. 3B). Consistent with the greater recovery efficiency of the ssDNA protocol, we find that most of the species detected in the dsDNA library preparation assays were also detected following ssDNA library preparation (95% species recovery, 934/984, Fig. 3C). 55% of all species detected were uniquely observed in the ssDNA library preparation assays.

The greater efficiency in recovery of microbial cfDNA is in line with the greater sensitivity of the ssDNA library preparation protocol to short fragment cfDNA. This feature offers the potential to profile the bacterial and viral components of the microbiome in plasma more effectively and to perform infectious disease diagnostics based on sequencing of cfDNA with increased precision and at lower cost. The ssDNA library preparation detected viral fragments with clinical relevance in transplantation, including polyomaviruses (BK Polyomavirus, one sample, and Merkel cell Polyomavirus, two samples) and single-stranded DNA, transfusion-associated viruses (including torque teno virus, 18 samples, and SEN virus, 19 samples); the impact of these viruses on the outcome of solid organ transplant patients has been investigated previously^15^. Current methods to detect infections are predominantly limited to testing one pathogen at a time^21^. Metagenomics approaches have the potential to broadly screen for all known pathogens (with a DNA genome) in a single test^13^,^15^,^22^,^23^. Blood can be collected non-invasively and the majority of tissues in the body are connected to the blood circulation, making cfDNA an attractive sample type for such approach. A number of caveats remain; for example, it may be difficult to inform about an infection by organisms that are part of the normal flora in certain body sites, but are pathogenic in others. cfDNA may be of limited use in such cases as it lacks body-site specificity.

### Donor-specific cfDNA

**Figure 4:**
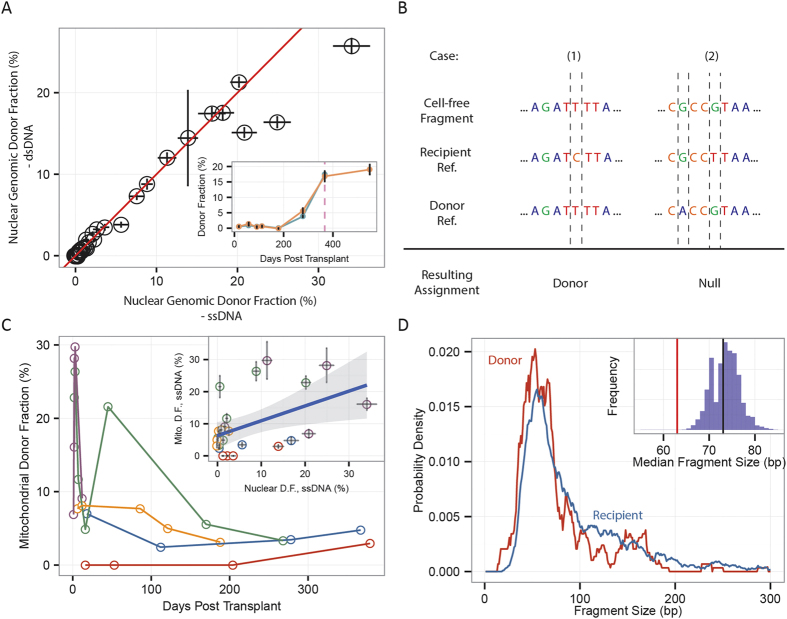
Quantifying donor-specific nuclear genomic and mitochondrial cfDNA. (**A**) Comparison of the fraction of donor-specific nuclear genomic DNA measured after dsDNA and ssDNA library preparation. In the inset, fraction of cfdDNA as function of time after transplant for a patient who suffered a severe acute rejection event at month 12 (cfdDNA measured by ssDNA (orange) and dsDNA (blue) library preparation). (**B**) Schematic representation of analysis workflow used to discriminate donor and recipient specific mt-cfDNA. Examples of an ambiguous assignment and a fragment assigned to the donor are shown. (**C**) Fraction of donor-specific mt-cfDNA as function of time post-transplant for five double lung transplant patients (25 samples, having excluded samples with fewer than 20 informative mitochondrial fragments); the inset compares the fraction of donor-specific mitochondrial and nuclear genomic DNA for the same samples (corr. = 0.463, Pearson, p = 0.0196). (**D**) Smoothed (distribution five nearest-neighbors, running mean) of donor mt-cfDNA is compared to the smoothed distribution of recipient mt-cfDNA. Inset: median fragment size for the donor mt-cfDNA compared to the fragment size of 10,000 subsets sampled from the recipient mt-cfDNA length set.

Donor specific cfDNA is present in the circulation of organ transplant recipients^2^ and recent studies have shown that the proportion of donor specific cfDNA (cfdDNA) is predictive of acute rejection in heart and lung transplantation^4^,^13^,^24^. We compared the fractional abundance of cfdDNA in the lung transplant samples measured following dsDNA and ssDNA library preparation (36 matched samples, six patients, Fig. 4A). We found an excellent agreement between matched measurements (corr. = 0.980, Pearson, p 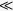 10^−5^). Here, sequences were assigned to the donor and recipient based on genotypic information (single-nucleotide polymorphisms, SNPs) obtained from pre transplant whole blood samples^13^. One patient (L36) suffered from a severe rejection event at month 12 post-transplant. The fraction of donor DNA measured for this patient using both library preparation methods was elevated coinciding with the biopsy-proven rejection event (Fig. 4A, inset).

Because of the high copy number of mtDNA in cells and the relatively high genetic diversity between two unrelated individuals^25^,^26^, mtDNA is often used in forensic analyses^27^ and in studies of population genetics^28^. The same attributes make mitochondrial DNA a promising candidate marker of post-transplant graft injury. We therefore asked whether donor-specific mitochondrial DNA can be detected in the plasma of transplant recipients. We built mitochondrial reference sequences to assign mitochondrial cfDNA to the transplant donor or recipient. To this end, DNA was extracted from whole blood samples collected from the donor and the recipient prior to the transplant procedure. Mitochondrial DNA was selectively amplified and sequenced. One million sequences led to a per-base coverage greater than 100-fold (genome size 16.5 kb), sufficient to determine subject-specific mitochondrial variants. Based on the reference sequences, we compiled lists of SNPs that are unique to either the donor or recipient (Fig. 4B, see Methods). On average, 152 informative SNPs were found per donor-recipient pair, roughly leading to a SNP every 114 bp. For samples prepared via ssDNA ligation, 8.7% ± 3.4% of the mitochondrial sequences were informative, and 9.5% of the informative SNPs were assigned to the donor. Donor and recipient specific sequences spanned the entire mitochondrial genome (Fig. S1).

To the best of our knowledge, this is the first direct observation of graft derived mitochondrial DNA in the circulation of transplant recipients. We computed the fractional abundance of donor-specific mt-cfDNA as the number of donor-specific mt-cfDNA molecules divided by the total number of informative mt-cfDNA molecules. We studied the variability and time dependence of the levels of donor-specific mt-cfDNA. The fraction of donor-derived mt-cfDNA was elevated during the first month post-transplant (Fig. 4C), in keeping with previous observations of elevated levels of cfdDNA in heart and lung transplant recipients during the first few weeks post-transplant, in the absence of acute rejection^4^,^13^. The fraction of donor-specific mt-DNA was only modestly correlated (corr. = 0.480, Pearson, p = 0.0152) with the fraction of nuclear genomic DNA. Samples for which there were less than 20 informative mitochondrial fragments were removed (11/36 samples, see Fig. S2 for correlation as function of sample size). Deeper sequencing and an analysis of a greater set of samples will be needed to investigate the relationship between acute rejection and the release of mitochondrial DNA from the graft.

Previous studies have found differences in fragment lengths for fetal and maternal cfDNA^20^, tumor and somatic cfDNA^9^ and hematopoietic and non-hematopoietic cfDNA^6^. Here, we compared the length of mitochondrial fragments derived from the graft (n = 265) to those specific to the recipient (n = 1855, 40 samples). We generated 10,000 random subsamples of the total collection of recipient-specific fragments (subsampled to the total number of donor fragments detected, n = 265). We next computed the median lengths for the random subsamples and compared to the median length of donor fragments (inset Fig. 4D). We found that donor sequences were slightly shorter (−9 ± 3 bp) than recipient-specific mitochondrial sequences. This shortening in fragment length may be indicative of differences in the mechanisms of release, or differences in processes of degradation, of donor and recipient mt-cfDNA.

## Discussion

In this work, we have demonstrated that a ssDNA library preparation is sensitive to cfDNA of a broad range of types and lengths. Few studies have focused on ultrashort cfDNA (with lengths shorter than 100 bp) or cfDNA that is not derived from the nuclear genome, including mitochondrial and microbial derived cfDNA. Our present work indicates that these relatively overlooked forms of cfDNA provide a unique window into physiology.

We applied a ssDNA library preparation to the analysis of cfDNA in the plasma of lung transplant recipients. We report the first observation of graft-derived mitochondrial DNA in the plasma of these organ transplant recipients. Donor-derived mitochondrial cfDNA has not been investigated as a marker of acute rejection in solid-organ transplantation, but offers several advantages: (1) the mitochondrial genome is small and relatively straightforward to deeply characterize via sequencing, (2) the mitochondrial genome contains a great number of variants that enables differentiation of donor and recipient sequences, and (3) with thousands of copies of mitochondrial DNA present in every cell, mitochondrial cfDNA is abundant in plasma. Mitochondrial DNA has conserved similarities to bacterial DNA and contains inflammatogenic unmethylated CpG motifs^29^. It is therefore not surprising that mitochondrial DNA was identified as a powerful damage associated molecular pattern–an endogenous molecule that can activate innate immunity when released during cellular injury^30^. It is conceivable that the release of mitochondrial DNA that accompanies graft injury promotes many of the harmful immunologic responses observed in solid-organ transplantation. The results presented here provide the first window into this relationship.

Microbial cfDNA is present in the circulation and is the product of microbial degradation across the body, or originates from microorganisms that infect the blood or translocate to the blood^31^. We found that the ssDNA library preparation is more effective at recovering bacterial and viral cfDNA, as compared to a dsDNA library preparation method. We furthermore found that the fragmentation profiles of microbial and mitochondrial DNA in plasma are highly similar, indicating that they are exposed to similar degradation processes. These observations enable measurement of the bacterial and viral microbiome in plasma with greater sensitivity and at a reduced cost.

Previous studies of the molecular size of nuclear genomic cfDNA have provided insight into the origin and nature of these molecules^32^. Many studies have noted that the predominant fragment size of cfDNA is consistent with the size of DNA wrapped around a single histone octamer^33^. Distinct length profiles are observed for cfDNA depending on their cellular origin with hematopoietically derived DNA being longer than that of nonhematopoietically derived^20^. Here, we found that the sequencing library preparation method can have a significant effect on length profile measurements. We report the fragmentation profile of nuclear genomic cfDNA in plasma over a broad range of lengths, and we conclude, in agreement with a recent report^33^, that a considerable fraction of nuclear genomic cfDNA is non-nucleosomal and subject to degradation by nucleases, in much the same way that we described for mitochondrial and nuclear genomic cfDNA.

In its current implementation, the ssDNA ligation requires more hands-on time compared to standard protocol (~13 hours versus ~6 hours, for 12 samples) at a similar cost per sample ($35–$40). This work focused on the cfDNA in plasma, but the methods described herein will further be relevant for genomic measurements of cfDNA in urine^20^. The widespread interest in circulating cfDNA as a marker of disease, warrants further investigation into the properties, types and origins of cfDNA and motivates further advances in genomic measurement techniques.

## Methods

### Study design and sample collection

We performed additional analyses and experiments on samples collected from six double-lung transplant recipients in the scope of a previous study^13^,^15^. Twelve whole blood samples collected prior to transplantation and 40 plasma DNA samples collected longitudinally after transplantation were analyzed.

### cfDNA sequencing assays

ssDNA sequencing libraries were prepared from cfDNA purified from plasma following a paleogenomic-based protocol^12^ with the following exceptions: (1) uracil excision steps using endonuclease VIII were not performed, (2) the amount of CircLigase II enzyme in the protocol was reduced from 4 μL to 0.8 μL and amounts of MnCl_2_ and CircLigase II buffer were halved, (3) editing of oligos including extension primer CL9, had an addition N*N*N*N overhang on the 5′ end (described by Karlsson *et al*.^17^) to prevent formation of adapter-dimers. A positive control (1 μL of 500 μM, synthetic ssDNA) and a negative control were included with each batch of samples. The efficiency of ligation of cfDNA fragments to biotinylated probes and ligation of double stranded adapters to primer-extended products was estimated using quantitative PCR^12^. On average 0.8 × 10^9^ unique ssDNA molecules (0.02–8.2 × 10^9^) were ligated and PCR amplified (8 to 15 cycles). Adjusting the extension sequence primer with a 4-N overhang on the 5′ end (as in Karlsson *et al*.^17^), limited the occurrence of adapter dimers to, on average, one in 1,700 sequences. Libraries were sequenced on the Illumina MiSeq or HiSeq platform (2 × 75 bp, average human genome coverage of 0.23x ± 0.06x). The list of edited oligos is presented in Supplementary Table 3.

### Mitochondrial reference sequences

Mitochondrial consensus sequences were established for every transplant donor and recipient. DNA was extracted from whole blood samples (Qiagen DNeasy Blood & Tissue kit) collected pre-transplant. Mitochondrial DNA was selectively amplified (Qiagen REPLI-g Mitochondrial DNA Kit), and sheared to 300 bp (Covaris). Libraries were prepared for sequencing using the NEBNext Ultra library preparation, characterized (Advanced Analytical Fragment Analyzer and dPCR) and sequenced (2 × 250 bp, Illumina MiSeq). One million sequences led to a per-base coverage greater than 100-fold (genome size 16.5 kb), sufficient to determine subject-specific mitochondrial variants. Fastq files were trimmed (Trimmomatic^34^, LEADING:25 TRAILING:25 SLIDINGWINDOW:4:30 MINLEN:15) and aligned against the human reference genome [GenBank:GCA_000001305.2] using BWA-mem^35^. Sequences that mapped to the mitochondrial reference sequence (edited from [GenBank:NC_012920) were extracted. A BCF file of SNPs was created and a FASTA consensus sequence was determined. A list of informative SNPs was created.

### Fragment length measurements by dPCR

We determined the abundance of mitochondrial and nuclear genomic cfDNA fragments of various sizes via dPCR (QuantStudio). Whole blood was obtained from a dog and cfDNA was isolated. A panel of amplicons for various sizes from 49 to 304 bp was created using IDT Custom Oligonucleotide Synthesis (see SI for primer design). Forward and reverse primers (each 10 μM, 0.3 μL) and cfDNA (2 μL) were mixed with 3.4 μL H_2_O, 7.5 μL QuantStudio 3D Digital PCR Master Mix (2X) (ThermoFisher Scientific, Cat. No. 4485718), and 1.5 μL of SYBR Green PCR Master Mix (ThermoFisher Scientific, Cat. No. 4309155). We performed PCR under the following conditions: (1) 96 °C for 10 min, (2) 55 °C for 2 min, (3) 98 °C for 30 sec, (4) repetition of (2)-(3) for 39 cycles, (5) 55 °C for 2 min, (6) 10 °C hold.

### Donor DNA measurements

Nuclear genomic sequences were assigned to the donor or recipient using methods previously described^4^,^14^. Briefly, sequences were assigned to the donor or recipient based on SNP genotyping information (IlluminaHumanOmni2.5–8 or HumanOmni1 whole genome arrays) obtained from pre-transplant whole blood samples. Mitochondrial sequences were assigned to the donor and recipient as follows: Raw sequencing datasets were trimmed (Trimmomatic^34^, LEADING:20 TRAILING:20 SLIDINGWINDOW:4:20 MINLEN:25), and low quality reads were filtered (FASTX toolkit^36^, -q 21 -p 50) and aligned (BWA-mem^35^) to the human reference genome [GenBank:GCA_000001305.2], with changes made to the mitochondrial genome (see SI methods). Sequences that mapped to the mitochondrial reference [GenBank:NC_012920] were collected and SNPs were listed using SAMtools^37^. Sequences were assigned to the donor or recipient through comparison to the list of informative SNPs compiled for the donor-recipient pair.

### Abundance of microbial cfDNA

The analysis workflow used to quantify non-human derived sequences is described in detail^13^,^15^. Briefly, Low-quality bases and Illumina specific sequences were trimmed (Trimmomatic 0.32^34^), and read pairs were merged using FLASH 1.2.7^38^. Reads were aligned (Bowtie 2.1.0^39^; very sensitive mode) against the human reference (UCSC hg19; https://genome.ucsc.edu/). Unaligned reads were extracted and BLASTed (2.2.28+) against a NCBI database^40^. Alignments were required to have an identity of at least 90% across 90% of the bases of the query. A relative genomic abundance of species was determined using GRAMMy^41^.

### cfDNA fragment sizing

Paired-end sequences were aligned (BWA-mem^35^) and the insert lengths were deduced from the coordinates of the bases at the outermost ends of each sequence pair. For microbial sequences, fragments assigned to microbial genomes using BLAST^40^, were retrieved and realigned using BWA-mem to determine the lengths of the library inserts.

### Statistical Analysis

All statistical tests were performed in R^42^, version 3.1.2.

### Ethics statement

All human-derived blood samples were collected from transplant patients at the Stanford University Hospital, who provided written informed consent in accordance with the guidelines under the approval of the Stanford University Institutional Review Board (Protocol 17666). The experimental protocols were approved under the Board’s review process. Mitochondrial consensus building, library preparation of cfDNA, cfDNA sequencing, and analysis were performed at Cornell University (#1412005225). All experimental methods were carried out in accordance with approved guidelines. All animal samples (canine-derived, whole blood) were collected from animals under care of the Cornell University Hospital for Animals and the Cornell University College of Veterinary Medicine (IACUC #2005-0151, Amendment #0001). All experimental protocols were approved by a committee of professionals and surgeons at the College and all methods were carried out under the accordance of their guidelines.

## Additional Information

**Accession codes:** Sequencing data are available in the Sequence Read Archive, BioProject PJRNA306662 (http://www.ncbi.nlm.nih.gov/bioproject/PRJNA306662).

**How to cite this article**: Burnham, P. *et al*. Single-stranded DNA library preparation uncovers the origin and diversity of ultrashort cell-free DNA in plasma. *Sci. Rep*. **6**, 27859; doi: 10.1038/srep27859 (2016).

## Supplementary Material

Supplementary Information
