# Supplementary material for: Single-stranded DNA library preparation uncovers the origin and diversity of ultrashort cell-free DNA in plasma
Source: Sci Rep. 2016 Jun 14;6:27859. doi: 10.1038/srep27859 (PMC4906518; doi:10.1038/srep27859)
Supplement: Supplementary Information [file srep27859-s1.pdf]

Supplementary Information for:

Single-stranded DNA library preparation uncovers the origin and diversity of ultrashort cell-free DNA in plasma

Authors: Philip Burnham[1], Min Seong Kim[1], Sean Agbor-Enoh[2], Helen Luikart[3], Hannah A. Valantine[2], Kiran K. Khush[3], Iwijn De Vlaminck[1,*]

1 Meinig School of Biomedical Engineering, Cornell University, Ithaca, NY 14853

2 National Institutes of Health, Bethesda, MD 20892

3 Division of Cardiovascular Medicine, Stanford University School of Medicine, Stanford CA 84305

*to whom correspondence should be addressed: id93@cornell.edu

File includes:

Supplementary text

Supplementary Tables 1-3

Supplementary Figures 1 and 2

References

Supplementary text

Mitochondrial Reference Sequence

Two changes were incorporated in the rCRS human mitochondrial genome [GenBank:NC_012920]. First, the reference base at position 3107[1] was replaced with an N. Second, the first 631 base pairs of the mitochondrial reference sequence were repeated at the end of the FASTA sequence, making the full reference 17,200 bp in length to represent the circular topology of the mitochondrial genome.

Calculating the Genomic Abundance from digital PCR Data

The abundance of mitochondrial and nuclear genomic DNA was assessed based on digital PCR assays with variable amplicon length (49-304 bp). The fraction of short fragments of DNA detected in PCR increases with decreasing amplicon size: the relative fraction of target molecules of length x, detected in a PCR assay with amplicon of length L, $f(x, L)$, can be calculated as: $f(x, L) = \frac{x-L+1}{x}$ for $L \leq x$. The abundance of cfDNA measured with a PCR assay with amplicon length L, $A_{PCR}(L)$, depends on the fragment size distribution G(x) expressed in genome equivalents, and can be computed as $A_{PCR}(L) = \sum_{k=L}^{\infty} G(k) f(k, L)$. We fitted this model to the experimental data in Figure 2A to estimate the abundance of mitochondrial and nuclear genomic cfDNA, assuming a Gaussian distribution (centered at 165 bp, standard deviation 20 bp) for nuclear genomic cfDNA and an exponential decay function for mitochondrial cfDNA.

Supplementary Table 1 – Relative Abundance Increase from Direct Strain Comparisons

Domain	All	Bacteria	Virus	Eukaryote	Archaea
**Number of comparisons**	1151	1024	30	96	1
**Mean RAI**	71.32x	73.70x	26.63x	60.54x	12.80x
**Median RAI**	17.67x	16.53x	10.91x	40.19x	12.80x
**Maximum RAI**	3949x	3949x	134.9x	477.0x	12.80x
**Minimum RAI**	0.2771x	0.2771x	0.7174x	0.4799x	12.80x

Supplementary Table 2 – Canine Primer Oligos for Fragment Length Detection in dPCR

Genome (gene)	Amplicon Length (bp)	Forward Primer Sequence (5'-3')	Reverse Primer Sequence (5'-3')
Mitochondrial	49	TAA GTA GGG CTG GTT AAT GC	CAC ATC TGC ACT CAC GCA TT
Mitochondrial	61	TCT TCT TAA ACT ATT CCC TGA CAC C	GAT ACT GAC ATA GCA CAG TAG GG
Mitochondrial	66	TCT GCT ATC ACT CAC CTA CGA	ATT CCC TGA GAG CAG AAG ATA AG
Mitochondrial	76	TCA CAC ATA ACT GTG GTG TCA T	CGT AGG TGA GTG ATA GCA GAT TC
Mitochondrial	98	AAG CTC TTG CTC CAC CAT C	GAT ACT GAC ATA GCA CAG TAG GG
Mitochondrial	127	AAG CTC TTG CTC CAC CAT C	GAA GAA GGG TTT ACC TGG AGA TAC
Mitochondrial	198	GGG ACA TCT CGA TGG ACT AAT G	GCT ACA AGT TAT TTG ACT GCA TTA GG
Mitochondrial	245	ACT ATC ATG AAA CTA TAC CTG GCA	ATT CCC TGA GAG CAG AAG ATA AG
Mitochondrial	304	TAC GCG CGC AAG ACA TTA	AGA TAG ACT ACG AGA CCA AAT GC
Nuclear (IGF1)	70	CTG ATT ACA GAA GGG AGT GTA TAG TT	CCC ACT CAA AGC AAT GGT AAT G
Nuclear (IGF1)	91	CAG CCT ACT GAT ACT GCC TTT C	CCC ACT CAA AGC AAT GGT AAT G

Nuclear (IGF1)	134	CAG CCT ACT GAT ACT GCC TTT C	CAA AGG GAT ACA CGG ACT ACA A
Nuclear (IGF1)	177	GTC AGT TCT TAG TTG CCC TTT ATT G	TAC TCC CTT CTC CCT TGT TCT
Nuclear (IGF1)	258	CAC TCT CTT GGG TGT GAG AAC	CAA AGG GAT ACA CGG ACT ACA A

Supplementary Table 3 – Oligo List for ssDNA Library Preparation (Not from Gansauge and Meyer)

Gansauge and Meyer ID	Description	Oligo Sequence (5'-3')
CL9	Extension primer	GTG ACT GGA GTT CAG ACG TGT GCT CTT CCG ATC T*N*N* N*N
CL53	Double-stranded adapter 1	ACA CGA CGC TCT TC/3ddC/
CL57	Double-stranded adapter 2	/5Phos/GGA AGA GCG TCG TGT AGG GAA AGA G*T*G* T*A*
CL78	Single-stranded adapter	/5Phos/AGA TCG GAA GTT TTT TTT TT/3BioTEG/

* All other oligos provided in the paper were kept the same.

Supplementary Table 4 – List of Reagents and Non-standard Materials Used

Product	Manufacturer	Catalog #
SDS buffer 20% solution	Life Technologies	am9820
SSC Buffer, 20X solution	Life Technologies	am9770
FastAP Thermosensitive Alkaline Phosphotase	Life Technologies	ef0654
dNTP mix (25 mM each)	Life Technologies	r1121
Dynabeads MyOne Streptavidin C1	Life Technologies	65001
T4 DNA polymerase	Life Technologies	18005017
Tango buffer, 10X solution	Life Technologies	by5
T4 DNA ligase (and 10x buffer)	Life Technologies	15224017
Accuprime pfx DNA polymerase (and 10x reaction mix)	Life Technologies	12344024
Bst 2.0 DNA Polymerase 1600 units	New England Biolabs	M0537S
pUC19 vector (50 ug)	New England Biolabs	N3041S
NEBNext® Ultra™ DNA Library Prep Kit for Illumina®	New England Biolabs	E7370L
NEBNext® Multiplex Oligos for Illumina® (Index Primers Set 1)	New England Biolabs	E7335L
Tween 20	Sigma-Aldrich	274348-4L

PEG-4000	Sigma-Aldrich	81240-1KG
Tris-HCl buffer (pH 8.0)	VWR	10128-396
NaCl, 5M solution	VWR	82023-090
MinElute PCR Purification Kit	Qiagen	28004
DNeasy Blood & Tissue Kit	Qiagen	69504
REPLI-g Mitochondrial DNA Kit	Qiagen	151023
QIAamp Circulating Nucleic Acid Kit	Qiagen	55114
Quality BIO 0.5M EDTA (pH 8.0)	Neta Scientific	QB-351-027-721EA
CircLigase II ssDNA ligase (and reaction buffer, $MnCl_2$)	Epicentre	CL9025K
iTaq™ Universal SYBR® Green Supermix	BioRad	1725121
QuantStudio™ 3D Digital PCR 20K Chip Kit v2 and Master Mix	ThermoFisher Scientific	A26317

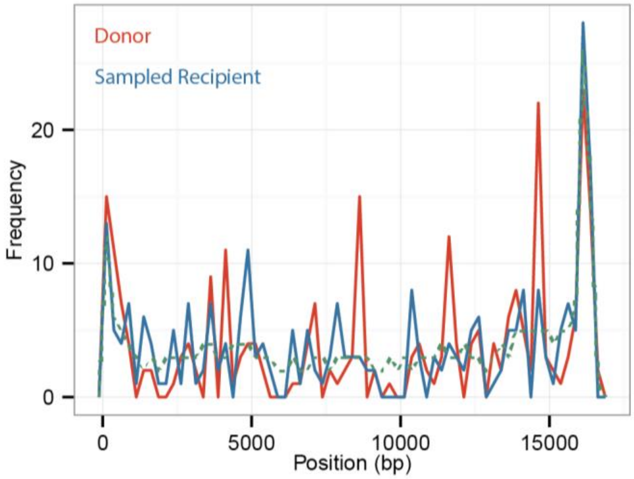

Figure S1: Comparison of Donor and Recipient Mitochondrial Cell-Free DNA. Analysis of the mitochondrial genomic positions from the donor and recipient are shown, no significant differences were observed[2].

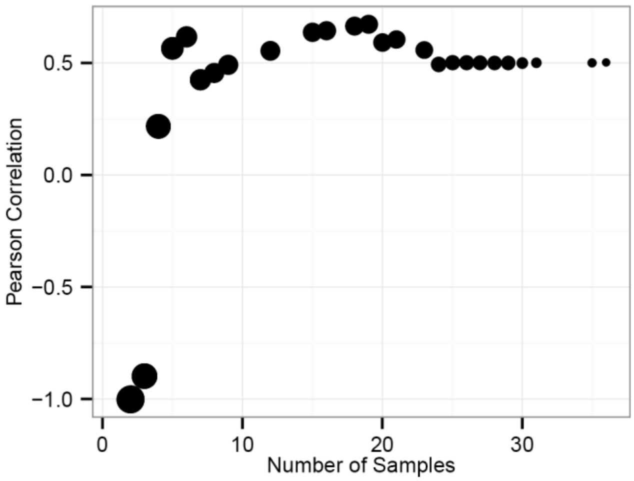

Figure S2: Correlation of Mitochondrial Donor Fraction with Filtering. Though many samples lacked a large number of informative mitochondrial SNPs, removal of these samples by increasing the minimum number of informative SNPs (size of data point) does not greatly alter the correlation of parameters based on the sample number.

Supplementary References

1. Andrews, R. M. *et al.* Reanalysis and revision of the Cambridge reference sequence for human mitochondrial DNA. *Nat Genet* **23,** 147 (1999).

2. Tsuji, J., Frith, M. C., Tomii, K. & Horton, P. Mammalian NUMT insertion is non-random. *Nucleic Acids Res.* **40,** 9073–88 (2012).
